# *Deconfounding Sex and Sex of Partner in Mate-Preference Research

**DOI:** 10.1177/09567976251319038

**Published:** 2025-03-11

**Authors:** Ashley J. Coventry, Selina Mixner, Benjamin Gelbart, Kathryn V. Walter, Daniel Conroy-Beam, Tamsin C. German

**Affiliations:** Department of Psychological and Brain Sciences, University of California, Santa Barbara

**Keywords:** evolutionary psychology, mate preferences, mating psychology, open data, open materials

## Abstract

Much of the previous research examining sex differences in human mate preferences has relied exclusively on heterosexual participants. Consequently, prior work overlooks a critical limitation: In heterosexual populations, participant sex and partner sex are perfectly confounded. Here, we tease apart this fundamental problem by separately examining ideal preferences for male and female partners across two studies—one using a large bisexual sample (*n* = 442) and another using a sample of both bisexual and heterosexual participants (*n =* 380). The results revealed that sex differences in mate preferences were largely driven by the participants’ own sex. However, both males and females set higher standards overall for the traits of male partners. These findings suggest that a person’s mate-preference psychology is shaped by both one’s own sex and the sex of the target being evaluated. More broadly, these results expand our understanding of the proximate psychology underlying human mate preferences.

An extensive body of research has sought to understand romantic- and sexual-partner preferences in humans. A large subset of this research examines sex differences (e.g., Buss, 1989; Li & Kenrick, 2006; Walter et al., 2020), with researchers identifying consistent sex differences in preferences for traits such as financial prospects, age, and physical attractiveness in long-term romantic partners. Although this research has been extremely valuable, it largely overlooks a critical confound: When studying exclusively heterosexual participants, participant sex is perfectly confounded with partner sex. Consequently, existing research has made it difficult or impossible to conclude whether preferences are determined by one’s own sex, the sex of the romantic target being evaluated, or the unique combination of own and target sex. Here, we addressed this confound by examining preferences for ideal male and female partners across two samples of bisexual participants. In so doing, this research allowed us to directly distinguish between preferences held *by* male and female participants and preferences held *for* male and female partners, as well as any interaction between the two.

Sex differences in mate preferences have long been a major focus of work on human mating. This work suggests that some mate preferences are sex-differentiated because human males and females have faced different adaptive problems throughout human evolutionary history (Buss, 1989). Males—more strongly limited in their ability to produce offspring by their ability to find and mate with fertile partners (Janicke et al., 2016)—should exhibit preferences for traits that are cues to fertility and reproductive value, such as physical attractiveness (Symons, 1979). On the other hand, females—limited in their ability to produce offspring by the large costs of gestation and lactation—should exhibit preferences for traits that serve as cues to a partner’s ability to procure and invest resources in those offspring and themselves (Symons, 1979). Additionally, parental investment theory predicts that the sex that has a greater parental investment burden will generally tend to be more selective in mate choice. In human romantic relationships, parental investment burdens are more equally shared than in many non-pair-bonding animals; nonetheless, given the large costs posed by gestation and lactation, we should expect if anything greater selectivity among females when choosing a mate relative to males (Kenrick et al., 1990; Trivers, 1972). Additionally, not all adaptive problems are unique to each sex. Males and females have shared adaptive problems in some domains and should thus share similar preferences for traits such as kindness, health, and intelligence (Buss, 1989).

Previous work supports these hypotheses. In long-term partners, males, relative to females, typically prefer ideal partners who are younger and higher in physical attractiveness (Buss, 1989; Hill, 1945; Lippa, 2007; Townsend & Wasserman, 1998; Walter et al., 2020). Females, relative to males, typically prefer ideal partners who are older and higher in ambition and financial prospects (Buss, 1989; Hill, 1945; Townsend & Wasserman, 1998; Walter et al., 2020). Moreover, across all preferences other than physical attractiveness, females around the world set higher ideal standards relative to males (Walter et al., 2020). However, both males and females more similarly value traits such as kindness, intelligence, and health (Lippa, 2007).

Although this work has been invaluable in identifying cross-cultural regularities in mate preferences, a major and largely unaddressed confound remains; namely, the vast majority of research on mate preferences has been conducted using exclusively heterosexual participants. This means that participant sex and partner sex have been perfectly correlated, generating a confound between the sex of the participant and the sex of the target to whom they are attracted (Fig. 1). For instance, heterosexual female participants are always asked about their preferences for a male partner, leaving researchers unable to disentangle whether these preferences are held because the participant is female or because these traits are sought after in male partners. For this reason, an important question remains unanswered: Are the “male-typical” and “female-typical” preferences identified in the prior literature truly tied to participants’ own sex—or are they a function of the sex of participants’ romantic or sexual target?

**Fig. 1.**
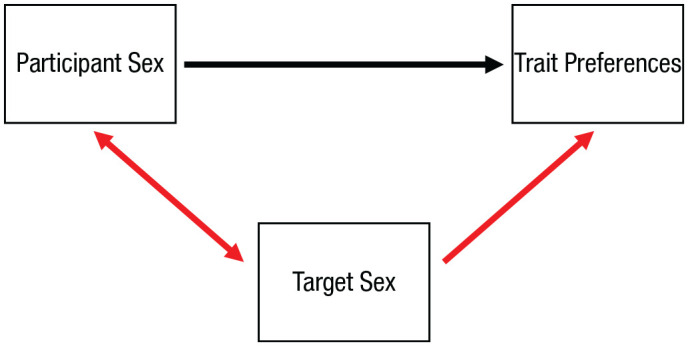
Confound of sex and partner sex on trait preferences. The black arrow depicts the currently established relationship between participant sex and trait preferences. The red arrows depict the heretofore untested role of target sex, which could instead be driving the relationship between participant sex and trait preferences. Among heterosexual participants, participant and target sex are perfectly correlated and therefore confounded in most prior work.

**Statement of Relevance**

Our partner choices have a large impact on our physical and mental well-being. Research dedicated to exploring the preferences we hold for these partners has found robust sex differences. However, much of this work has been conducted with exclusively heterosexual participants, making it impossible to determine whether these sex-differentiated preferences exist because males and females hold different preferences or because people, regardless of sex, prefer different traits in male and female partners. This work, examining preferences in bisexual and heterosexual participants, suggests that both one’s own sex and the sex of the partner are important in influencing preferences. Whereas individual sex differences in preferences were driven by participant sex, overall standards were driven by partner sex. This work highlights the importance of including nonheterosexual participants in relationship research and offers important implications for future research on bisexual partner preferences.

One notable exception to this body of research comes from Howard and Perilloux (2017), who used samples of heterosexual, gay, and lesbian participants to deconfound several dimensions of mating psychology, such as sociosexuality. Although the authors found evidence that these dimensions of human mating psychology do, in fact, appear to be more strongly tied to one’s own sex (rather than preferred partner sex), they did not examine mate preferences. Thus, it remains unclear how this finding applies to ideal trait preferences. Moreover, although including gay and lesbian participants deconfounds participant and partner sex, it still confounds partner sex and sexual orientation. For example, any differences between straight and gay men could be attributable to differences in the sex of their romantic target or to differences in their sexual orientation.

In contrast, bisexual participants present a more direct way to address the confound between one’s own sex and the sex of one’s partner. Because bisexual participants are attracted to both men and women, they afford an opportunity to directly disentangle the contributions of own sex and partner sex by using each participant, in essence, as their own control. Although prior research has examined preferences in bisexual participants, existing research has asked only about participants’ overall preferences for an ideal partner without separately assessing preferences for male partners and preferences for female partners (e.g., Dobersek et al., 2023; Klümper et al., 2024; March et al., 2015; Takayanagi et al., 2024). Here, we tested the possibility that preferences may shift as a function of target sex across two studies.

In Study 1, we assessed to what extent preferences differed as a function of participant sex or target sex in a sample of bisexual participants. In Study 2, we used a mixed sample of bisexual and heterosexual participants to replicate the results of Study 1 and assessed whether participant sexual orientation, separate from target sex, affected these preferences.

## Research Transparency Statement

### General disclosures

**Conflicts of interest:** All authors declare no conflicts of interest. **Funding:** This research was funded by a University of California, Santa Barbara Faculty Research grant to Daniel Conroy-Beam and Tamsin German. **Artificial intelligence:** No AI-assisted technologies were used in this research or the creation of this article. **Ethics:** This research received approval from the University of California, Santa Barbara Institutional Review Board. **Computational reproducibility:** The authors are not applying for a Computational Reproducibility Badge.

### Study disclosures

#### Study 1

**Preregistration:** This study was not preregistered. **Materials:** The materials are publicly accessible at https://doi.org/10.17605/OSF.IO/AMX5V. **Data:** The data are publicly accessible at https://doi.org/10.17605/OSF.IO/AMX5V. **Analysis scripts:** The analysis scripts are publicly accessible at https://doi.org/10.17605/OSF.IO/AMX5V.

#### Study 2

**Preregistration:** This study was not preregistered. **Materials:** The materials are publicly accessible at https://doi.org/10.17605/OSF.IO/AMX5V. **Data:** The data are publicly accessible at https://doi.org/10.17605/OSF.IO/AMX5V. **Analysis scripts:** The analysis scripts are publicly accessible at https://doi.org/10.17605/OSF.IO/AMX5V.

## Study 1

### Method

#### Participants

Participants were recruited as part of a large U.S. sample of LGBT participants recruited through Prolific and Qualtrics’s survey panel service. The study was advertised as “Project Rainbow America,” the purpose of which was described as examining “what characteristics people of all sexual orientations and genders like and dislike in a romantic partner.” Data were collected in waves; within each wave, the survey was made available to people only of the particular sexual or gender identity being targeted in that wave. Participants were asked to self-report their sexual and romantic identity, as well as the gender(s) they could imagine themselves having a long- and short-term relationship with. Participants were retained in the sample for this study and the analyses that follow if they reported that they could imagine themselves “having a long-term relationship with a man” and “having a long-term relationship with a woman” or if they reported that they could imagine themselves having a short-term relationship with both a man and a woman. We chose to include participants on the basis of these criteria instead of using their reported identity as bisexual because there are a multitude of different labels that describe romantic and/or sexual attraction to multiple genders (e.g., bisexual/biromantic, pansexual/panromantic, omnisexual/omniromantic), and we did not want to falsely eliminate participants who reported attraction to both sexes. Although 42 participants in our sample still self-identified as heterosexual and heteroromantic after applying those criteria, they were retained in this sample because of their reported sexual or romantic attraction to both men and women. Analyses excluding this subset of participants are available in the Supplemental Material available online; results generally parallel those discussed here. For simplicity, we use the term “bisexual” throughout this article as an umbrella term to refer to anyone that experiences attraction to both sexes. Additionally, because participant sex is necessary for our analyses, we also eliminated one participant who did not report their sex.

After applying these exclusion criteria, the final sample contained 442 so-identified bisexual participants (250 females, 56.56%; 192 males, 43.44%) whose mean age was 31.4 years (*SD* = 12.83). Of these participants, 36.65% reported that they were single. The remainder reported casually dating a partner (7.92%), seriously dating a partner (27.15%), being engaged to a partner (6.79%), or being married to a partner (21.49%).

#### Procedure and measures

This procedure adhered to the ethical guidelines set forth by the University of California, Santa Barbara Institutional Review Board. After providing informed consent, participants completed a mate-preference questionnaire adapted from Conroy-Beam et al. (2022) to refer separately to ideal male and female partners. The questionnaire referred to either short-term or long-term preferences depending on whether participants indicated short-term attraction, long-term attraction, or both in relation to each sex. For example, if participants reported short-term attraction to males and females but long-term attraction to only males they answered questions about their long-term preferences for male partners only, as well as items about their short-term preferences for male and female partners. In the survey, long-term relationships were described as committed, romantic relationships, and short-term relationships were described as uncommitted, sexual relationships. For brevity, we focus here on the analysis of the long-term preferences; results from the analyses of short-term preferences are described in the Supplemental Material. Results were overall similar for both sets of preferences.

Participants rated their preferences for five traits (health, intelligence, kindness, physical attractiveness, and financial resources) on a scale from 1 to 11 (1 = *bottom 1 out of 100 people*, 11 = *top 1 out of 100 people*). Two items were asked for each trait, and ratings were averaged to form composites. For example, to assess ideal preferences for resources in a long-term female partner, participants were asked both “How financially stable should your ideal female romantic partner be (i.e. your most preferred value)?” and “How good should your ideal female romantic partner’s earning potential be (i.e. your most preferred value)?” Scores on both of these items were averaged together to form a composite for ideal resource preference (per the procedure described in Conroy-Beam et al., 2022). A rating of *bottom 40 out of 100 people* for this trait would mean that a participant would prefer slightly below average resources in an ideal female partner. Participants were asked about their ideally preferred trait value for each dimension rather than the minimum standards or relative importance of each dimension because prior work has demonstrated that ideally preferred values are most directly linked to actual mate choices (Conroy-Beam et al., 2022).

Participants also reported their ideal age range for this partner on a scale from 1 to 11 (1 = *20 or younger*, 11 = *75 or older*) with 6-year intervals in between (e.g., 21–26, 27–32, 33–38). Both the order of these preference items as well as the order of the long-term and short-term preference blocks were counterbalanced.

To estimate our power to detect effects of interest given our sample size, we conducted a sensitivity analysis using the simr package in R (Green & MacLeod, 2016) based on the sensitivity analysis conducted by Jaeger (2021). We first determined the effect size we would be able to detect from a potential three-way interaction between participant sex, partner sex, and trait. With our sample size, results of the sensitivity analysis suggested we would have 80% power to detect an effect (*R*^2^) of approximately .0023 (estimated using the r2beta function in the r2glmm package; Jaeger, 2017). We furthermore determined the effect size we could detect for lower order effects, specifically the potential main effect of partner sex because this was the effect deemed to be of most interest. From the sensitivity analysis, we found we could detect an effect (*R*^2^) of approximately .0021 with 80% power. Because powering three-way interactions is rather difficult, findings concerning the three-way interaction should be interpreted with caution. Finally, we also determined the effect size we would be able to detect with 80% power for a potential two-way interaction between participant sex and target sex (recoded as same or opposite to the participant) on resource preferences. This was based on the results of our resource-preference analyses detailed in the Results section. Our simulations suggested we would have 80% power to detect an effect size (*R*^2^) of approximately .0072.

### Results

Participants’ ideal trait ratings were most often between 5 (*top 50 out of 100 people*) and 7 (*top 18 out of 100 people*). The mean rating for all traits was above average, or *top 50 out of 100 people*. Table 1 presents descriptive information of trait ratings for the five measured traits.

**Table 1.** Study 1: Descriptive Statistics of the Preference Items

Trait	Ideal preference ratings			
	*M*	*SD*	Minimum	Maximum
Health	6.02	1.55	1.00	10.00
Intelligence	6.65	1.63	0.50	10.00
Kindness	7.04	1.65	1.00	10.00
Physical attractiveness	6.03	1.58	0.50	10.00
Resources	6.02	1.52	0.00	10.00

Note: The traits described here are the composites of the two items assessing each trait. Age is not described here because it was measured on a different scale from the other traits.

To assess whether preferences varied as a function of one’s own sex or the sex of one’s romantic target, we first fit a linear mixed model predicting the ideal preference value as an outcome variable (i.e., the participants’ rating for each of the five measured traits) and trait dimension, target sex, and participant sex as predictor variables, nesting preferences within participants with a random intercept term. We then proceeded with a backward elimination strategy, first fitting a model with the three-way interaction between trait, target sex, and participant sex, removing any nonsignificant higher order interactions and repeating this process until only significant interactions remained.

The final model showed a significant main effect of target sex, *F*(1, 3446.2) = 14.768, *p* < .001, and a significant interaction between participant sex and trait, *F*(5, 3037.0) = 15.589, *p* < .001. The main effect of target sex was such that preference values were higher across dimensions for male targets than for female targets (*b* = 0.20), indicating that, across traits, both male and female participants held higher ideal standards for male partners.

**Fig. 2.**
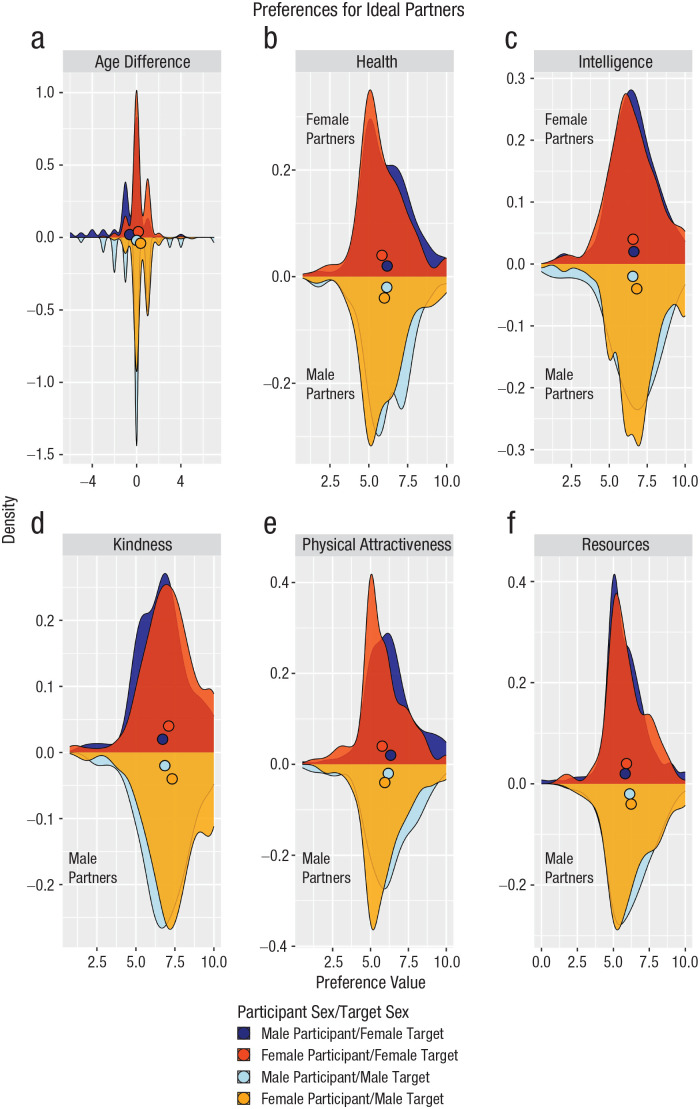
Distribution of preference ratings for long-term partners. Density plots of ideal preference values in a long-term partner are shown for (a) age difference, (b) health, (c) intelligence, (d) kindness, (e) physical attractiveness, and (f) resources. Preferences for female targets are shown above the *x*-axis, and preferences for male targets are mirrored below the *x*-axis; negative densities are merely the ordinary density multiplied by −1 for the purposes of mirroring. Age difference was set on a different scale from other traits, with 0 indicating no age difference between the participant and their ideal partner. Negative values indicate an ideal partner younger than the participant, whereas positive values indicate the opposite. The plots also contain mean points for each participant/target sex combination for each trait. All other traits were measured on a scale from 0 to 10.

To explore the interaction between participant sex and trait, we refit linear mixed models predicting each trait separately from the target sex and participant sex. Figure 2 shows the distribution of preference ratings for each trait for men and women when evaluating male partners/targets and when evaluating female partners/targets. These analyses (with the exception of age, which was measured on a different scale) were based on standardized trait values; thus, *b* values may be interpreted comparably to Cohen’s *d*. Male participants preferred ideal partners who were more physically attractive (*b* = 0.28, *p* = .004) and younger (*b* = −0.67, *p* < .001) relative to female participants, consistent with established research on sex differences in mate preferences. Additionally, male participants preferred ideal partners who were significantly less kind relative to female participants (*b* = −0.26, *p* = .007).

**Fig. 3.**
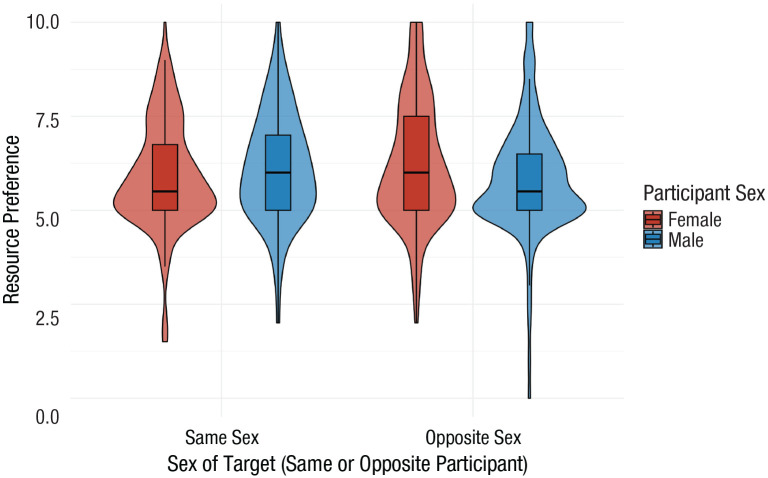
Resource preferences for same- and opposite-sex targets. The violin plots show the ideal resource-preference values in a long-term same-sex and opposite-sex partner. Ideal preferences for resources were measured on scale from 0 to 10.

Notably, we did not find a significant effect of participant sex on preferences for resources (*b* = −0.08, *p =* .389), seemingly in contrast with previous research finding a sex difference in resource preferences in heterosexual participants. To further determine whether the difference in results was driven by target sex, we used a linear mixed-effects model predicting resource preference from a potential interaction between participant sex and target sex, recoding target sex to refer to targets as either the same sex as the participant or the opposite sex of the participant. Here, we did find a significant interaction between participant sex and whether the target was of the same or opposite sex (*b* = −0.37, *p* = .004; Fig. 3). Preferences descriptively replicated prior research when considering opposite-sex targets: Female participants (*M* = 6.23) reported greater desire for resources in a partner than did male participants (*M* = 5.82). However, this pattern reversed when considering same-sex targets: Male participants (*M* = 6.13) reported greater on-average preferences for resources than did female participants (*M* = 5.91). Although this suggests that the long-standing sex difference in preferences for resources is robust, it also suggests that this sex difference may emerge as a result of target sex rather than participant sex.

Finally, we also ran an additional exploratory model that included relationship status as a predictor variable to determine whether relationship status significantly interacted with participant sex, target sex, or trait. These results are described in Supplemental Analyses 3 in the Supplemental Material and did not qualitatively differ from those reported here.

## Study 2

### Method

#### Participants

Participants were recruited through Prolific for a study listed as “Study on Relationship Preferences.” The listed purpose of the study was the same as for Study 1. We used the quota feature on Prolific to specify a sample evenly divided between males and females and between heterosexual and bisexual participants. We recruited 498 participants. Participants were eliminated if they failed the attention check (*n* = 35) or if they identified as something other than heterosexual or bisexual (based on the criteria of bisexuality described in Study 1; *n =* 83).

Our final sample consisted of 380 participants, of whom 198 (52.11%) were female (106 bisexual and 92 heterosexual) and 182 (47.89%) were male (76 bisexual and 106 heterosexual). Overall, 182 (47.89%) participants were bisexual, whereas 198 (52.11%) were heterosexual. Participants were slightly older than the participants in Study 1 (*M* = 37.47 years, *SD* = 11.53). Most participants were married (*n* = 151; 39.74%); however, 27.63% reported being single, 22.89% reported seriously dating, 5.00% reported casually dating, and 4.74% reported being engaged.

As in Study 1, we estimated a sensitivity analysis using the simr package in R to determine the smallest effect size we could detect with 80% power given our sample of bisexual participants (*n* = 182). Here, we focused on the potential three-way interaction effect between participant sex, partner sex, and trait. We found that we would be able to detect an effect (*R*^2^) of approximately .0037 with 80% power. Using the r2beta function in the r2glmm package in R, we were able to determine that the estimated effect size (*R*^2^) of the potential three-way interaction in Study 1 was approximately .0020. Thus, it was plausible that we would be able to detect a comparable effect size in this study given our sensitivity analysis. Furthermore, we determined that we would be able to detect a main effect (*R*^2^) of partner sex of approximately .0022 with 80% power.

We also conducted a sensitivity analysis based on the three-way interaction of participant sex, trait, and sexual orientation with our sample of bisexual and heterosexual participants (*n* = 380) and their opposite-sex preferences (for more details on the linear mixed-effect model analysis, see the Results section below). With our sample size, we were able to detect a three-way interaction effect (*R*^2^) of approximately .0037 with 80% power. As mentioned in detailing our Study 1 sensitivity analyses, these higher order interactions are difficult to power, and so caution should be used in interpreting the related findings. We were able to detect a two-way interaction between participant sexual orientation and trait, with the ability to detect an effect size (*R*^2^) of approximately .0037 at 80% power. Finally, we conducted a sensitivity analysis based on the potential two-way interaction between participant sex and target sex (recoded as same or opposite to the participant) on resource preferences. From this, we determined that we were able to detect an effect (*R*^2^) of .0050 at 80% power.

#### Procedure and measures

The procedure and measures were largely the same as those used in Study 1 except that the participants in Study 2 answered only long-term-preference questions. We also asked participants to specifically imagine a bisexual partner. We did this to address the possibility that, in the previous study, male and female participants may have been imagining different types of ideal partners; for example, female participants could have been picturing a male partner interested only in women, whereas male participants could have been picturing a male partner interested only in men. Furthermore, without specifying target sexual orientation, bisexual participants might consider partners with a range of sexual orientations, whereas heterosexual participants might consider only heterosexual partners.

Bisexual participants received preference questions about ideal male and ideal female partners separately, as described in the Procedure and Measures section for Study 1. Heterosexual participants received preference questions only about an ideal opposite-sex partner.

### Results

As in Study 1, participants’ ideal trait ratings were most often between 5 (*top 50 out of 100 people*) and 7 (*top 18 out of 100 people*), with an above average (*top 50 out of 100 people*) mean rating across all traits (for descriptive statistics, see Table 2).

**Table 2.** Study 2: Descriptive Statistics of the Preference Items

Trait	Ideal Preference Ratings			
	*M*	*SD*	Minimum	Maximum
Health	6.37	1.42	1.50	10.00
Intelligence	6.77	1.36	1.00	10.00
Kindness	7.17	1.48	1.50	10.00
Physical attractiveness	6.36	1.41	1.00	10.00
Resources	6.34	1.48	1.50	10.00

Note: The traits described here are the composites of the two items assessing each trait across both bisexual and heterosexual participants. Age is not described here because it was measured on a different scale from all other traits.

We first directly replicated the analyses performed in Study 1 using only bisexual participants. Specifically, we fit a linear mixed model predicting the ideal preference value (the ratings of each of the five measured traits) from a main effect of partner sex and an interaction between trait and participant sex, nesting preferences within participants with a random intercept term.

Replicating the results of Study 1, the model showed a significant main effect of target sex, *F*(1, 1976.1) = 11.70, *p* < .001, and a significant interaction between participant sex and trait, *F*(5, 1974.2) = 21.54, *p* < .001. The main effect of target sex revealed that preference values were higher for male targets than for female targets (*b =* 0.15), showing that participants—regardless of sex—again reported higher standards, overall, for male partners. To ensure that we did not miss a significant higher order effect, we fit a linear mixed model predicting the ideal preference value from the three-way interaction between participant sex, partner sex, and trait and did not find a significant effect, *F*(5, 1963.2) = 0.52, *p =* .765.

As in Study 1, we also ran an additional model including relationship status as a predictor variable to determine whether relationship status significantly interacted with these effects. These results are detailed in Supplemental Analyses 4 in the Supplemental Material and did not change our overall findings.

**Fig. 4.**
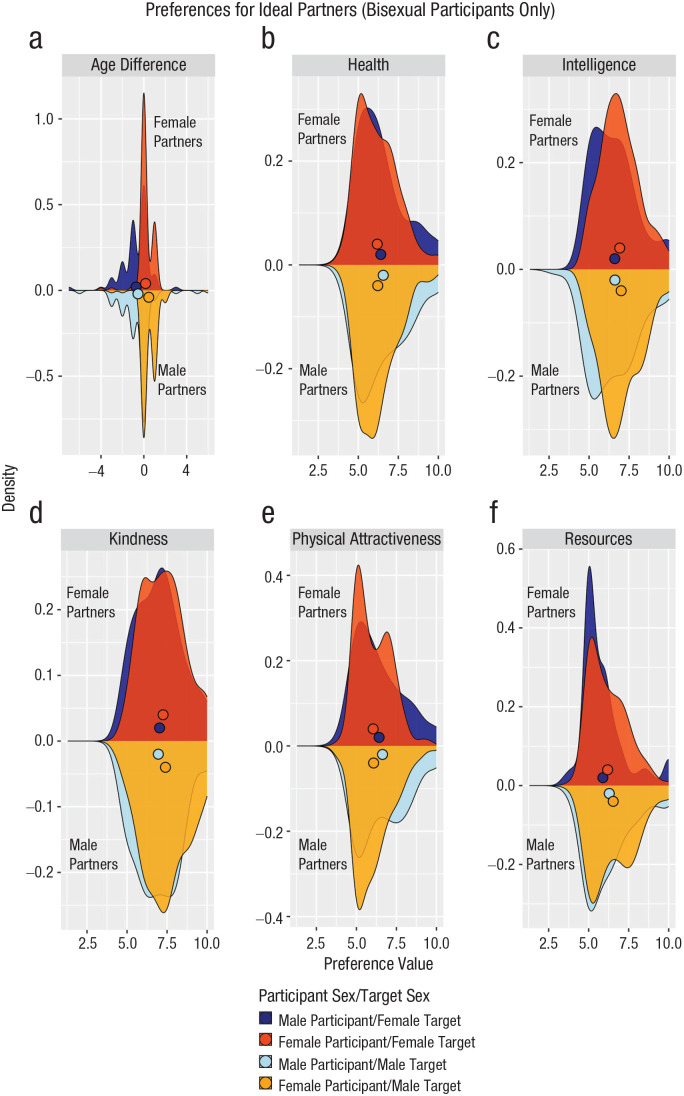
Distribution of bisexual participants’ preference ratings for long-term partners. Density plots of ideal preference values for each trait in a long-term partner are shown for (a) age difference, (b) health, (c) intelligence, (d) kindness, (e) physical attractiveness, and (f) resources. Preferences for female targets are shown above the *x*-axis, and preferences for male targets are mirrored below the *x*-axis; negative densities are merely the ordinary density multiplied by −1 for the purposes of mirroring. Age difference was set on a different scale from other traits, with 0 indicating no age difference between the participant and their ideal partner. Negative values indicate an ideal partner younger than the participant, whereas positive values indicate the opposite. The plots also contain mean points for each participant/target sex combination for each trait. All other traits were measured on a scale from 0 to 10.

We explored the participant sex-by-trait interaction by refitting linear mixed models predicting each trait separately from target sex and participant sex. As in Study 1, these analyses (with the exception of age, which is on a different scale) were based on standardized trait values, so *b* values can be interpreted comparably to Cohen’s *d*. Replicating Study 1, results showed that male participants, relative to female participants, preferred more physically attractive (*b* = 0.35, *p* = .012) and younger (*b =* −0.95, *p <* .001) ideal partners. However, unlike Study 1, male and female participants’ overall preferences for kindness (*b* = −0.25, *p* = .085) were not significantly different. Figure 4 shows the distribution of preference ratings among bisexual participants for each trait among men and women separated by partner sex.

**Fig. 5.**
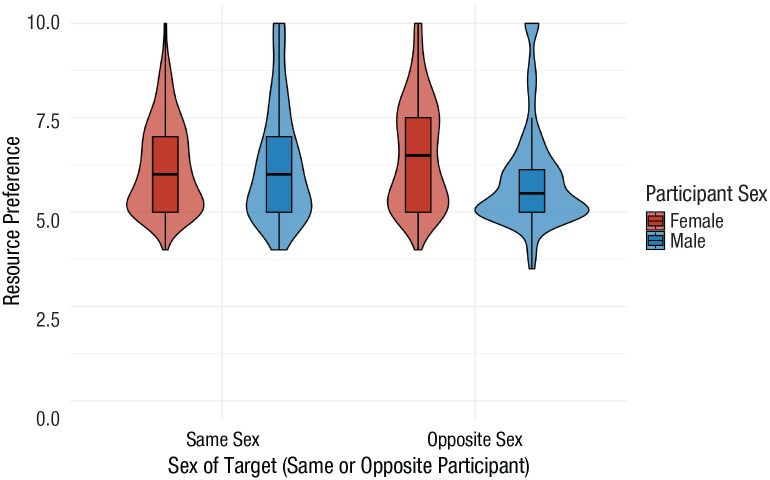
Resource preferences for same- and opposite-sex targets. The violin plots show the ideal resource-preference values in a long-term same-sex and opposite-sex partner. Only bisexual participants are included. Heterosexual participants did not complete ideal preference measures about same-sex targets and thus were excluded from this analysis. Ideal preferences for resources were measured on a scale from 0 to 10.

Consistent with our findings in Study 1, there was no significant difference between male and female participants in preferences for resources (*b* = −0.20, *p =* .165). We again explored this deviation from findings in previous work with an additional model predicting resource preference from a potential interaction between participant sex and target sex, with target sex recoded as same as or opposite of participant sex. Here, as in Study 1, there was a significant interaction between participant sex and whether the target was the same or opposite sex (*b* = −0.54, *p* < .001; Fig. 5). When considering opposite-sex targets, preferences descriptively replicated prior research: Female participants (*M* = 6.53) reported greater desire for resources in a partner than did male participants (*M* = 5.89). However, when considering same-sex targets, this pattern reversed: Male participants (*M* = 6.19) reported greater on-average preferences for resources (*M* = 6.29) than did female participants.

**Fig. 6.**
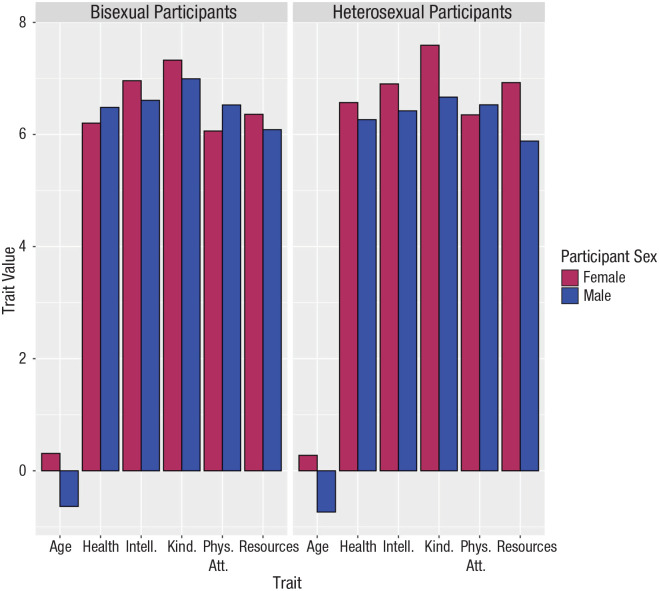
Bisexual and heterosexual participants’ preference ratings for long-term partners. Ideal preference values held by males and females are shown for each trait faceted by sexual orientation. Age difference was set on a different scale from other traits, with 0 indicating no age difference between the participant and their ideal partner. Negative values indicate an ideal partner younger than the participant, whereas positive values indicate the opposite. All other traits were measured on a scale from 0 to 10.

One potential limitation of Study 1 is that bisexual participants may have fundamentally different mate preferences than do heterosexual participants, and so sex-difference findings concerning their preferences may not generalize to heterosexual participants. To address the possibility, we also reran our analysis with both bisexual and heterosexual participants, looking only at opposite-sex preferences, and examined a potential effect of sexual orientation. We predicted ideal preference values from an interaction of trait, participant sex, and sexual orientation, once again nesting preferences within participants with a random intercept term. The three-way interaction between participant sex, sexual orientation, and trait was not significant, *F*(5,1775) = 1.90, *p* = .091. However, because this interaction could be interpreted as marginally significant, and because such a three-way interaction could limit the generalizability of results from bisexual participants to heterosexual participants, to be maximally conservative we elected to more fully explore this interaction effect. To do this, we decomposed the three-way interaction of participant sex, sexual orientation, and trait by exploring the interaction between participant sex and sexual orientation for each trait using standardized trait values. We found only a significant interaction between participant sex and sexual orientation in predicting preferences for kindness, *F*(3, 355) = 9.62, *p* = .021. This interaction was such that sex differences were larger for heterosexual participants (*M*_females_ = 7.66, *M*_males_ = 6.59) relative to bisexual participants (*M*_females_ = 7.40, *M*_males_ = 7.03) when considering the preferred kindness of a potential partner. No other significant interactions between participant sex and sexual orientation were observed. Overall, even if the three-way interaction between participant sex, trait, and sexual orientation were considered genuine, preferences were largely the same between heterosexual and bisexual participants (see Fig. 6).

Finally, we ran all tests again using an integrative approach. We pooled our bisexual samples from Study 1 and Study 2 together and reran our linear models in an attempt to detect any potential higher order effects we may not have been able to observe with our smaller, individual samples. In so doing, we replicated our previous results and found no significant higher order effects. Details of these analyses are listed in Supplemental Analyses 5 in the Supplemental Material.

## General Discussion

What humans prefer in an ideal partner represents one of the largest areas of research in relationship science. Nonetheless, much of this research is constrained by a critical confound: Because males are typically evaluating ideal female partners and females are typically evaluating ideal male partners, the role played by participant sex is indistinguishable from the role played by target sex. Here, we addressed this confound by examining sex differences in preferences in two samples: one exclusively comprising bisexual participants and another comprising both heterosexual and bisexual participants.

Across both samples, sex differences in ideal preferences for age and physical attractiveness of long-term partners appeared to be driven by one’s own sex rather than the sex of the target. This is consistent with previous work on sex differences in preferences (Buss, 1989; Walter et al., 2020). By contrast, preferences for resources did not appear to be driven by participant sex. Rather, this classic sex difference appears to have been carried, at least in part, by a difference in overall standards driven across targets: Both male and female participants ideally preferred higher levels of all traits in male partners relative to female partners. This newly discovered difference in ideal standards as a function of target sex offers a partial explanation for some previously documented sex differences that is not observable in exclusively heterosexual samples.

Although we did find a marginally significant interaction between sexual orientation, participant sex, and trait in predicting preferences, we found a significant trait-level interaction between sexual orientation and participant sex only for kindness preferences, in which sex differences increased among heterosexual relative to bisexual participants. Otherwise, however, preferences appeared to be largely the same between heterosexual and bisexual participants. This suggests that these results are unlikely to reflect a unique phenomenon tied to bisexuality and point to the possibility that overall standards may be one aspect of mating psychology that is tied to the evaluations of male partners—rather than the psychology of the evaluator’s sex.

Overall, these results also suggest that we have been missing a full understanding of mating psychology by ignoring a major confound in mating-psychology research. Parental investment theory suggests that the sex investing more heavily in offspring should be more selective when evaluating potential mates (Trivers, 1972). In humans, this has been largely assumed to mean that females are more selective, and set higher standards, when evaluating potential mates (e.g., Kenrick et al., 1990). In a similar vein, sexual strategies theory posits that sex-specific mate preferences result from unique adaptive challenges faced by males and females in ancestral environments (Buss & Schmitt, 1993).

However, whereas both of these theories make clear predictions about the expected functional outcomes of human mating psychology—for instance, females preferentially mating with males possessing cues to good financial prospects—multiple proximate avenues may achieve this functional end. Existing research has commonly assumed that sex differences in preferences reflect a sexually dimorphic psychology. However, our results suggest that sex differences in mate preferences could be driven, in part, by a sexually *monomorphic psychology* in a sexually *dimorphic context*: evaluating partners who differ in their sex. These results highlight the importance of considering not only observed, manifest outputs in theoretical analyses but also the possible proximate designs through which these manifest outputs may be generated (Cosmides & Tooby, 1987).

Despite these strengths, the current research does have some limitations. First, in using results from bisexual samples to draw conclusions about the design of human mating psychology, we are generalizing results from bisexual participants to heterosexual populations. It is possible, however, that our results may be affected by participant sexual orientation. Although overall trends remained the same between our heterosexual and bisexual samples, we did find a marginally significant interaction involving sexual orientation and a significant effect between sexual orientation and participant sex on ratings of kindness specifically. Additionally, that the classic sex difference in resource preferences replicated only when analyses were restricted to opposite-sex targets could be interpreted as evidence that this preference was entirely driven by partner sex or that we were underpowered to detect a participant-sex difference. Based on the sensitivity analyses, there was sufficient power to detect small to medium effect sizes, and thus a smaller participant-sex effect may have been missed. Furthermore, three-way interactions are much harder to power, and thus it is possible that the interaction effect was smaller than was possible to detect. Results surrounding three-way interactions should be interpreted with caution, and future research should explore the relationships between sexual orientation and mating psychology using larger samples.

Second, a main limitation in this work is that participants across both studies were exclusively recruited in the United States. Although many of the preferences we examined have been replicated in a wide range of cultures (Atari et al., 2020; Buss, 1989; Butovskaya & Smirnov, 2005; Marlowe, 2004; Scelza & Prall, 2018; Walter et al., 2020), considerable cross-cultural variability in preferences also exists (e.g., Walter et al., 2021). Future research could address this limitation by examining cultures outside of the United States.

An additional limitation to consider is that although participants were instructed to think of bisexual ideal partners in Study 2 (to ensure bisexual participants were imagining male and female partners of the same sexual orientation), it is possible that biases in perceptions of bisexual men and women led to different considerations of ideal male and female partners. For example, Morgenroth et al. (2021) found evidence suggesting that bisexual men are viewed as more attracted to men than to women, whereas bisexual women are not viewed preferring one gender over the other.

Future research could also expand the current work to examine how these preferences manifest in the context of actual mate pursuit. In existing research on preferences, a large body of research has centered around the extent to which stated mate preferences correspond to actual mate choices (Conroy-Beam & Buss, 2016a, 2016b; Eastwick & Finkel, 2008; Eastwick et al., 2011). In light of these disagreements, extending the current work to real-life choice contexts may be particularly fruitful.

## Conclusion

This study examined whether mate preferences are primarily driven by one’s own sex or the sex of one’s romantic target. To our knowledge, this study is the first in the literature to separately assess bisexual participants’ preferences for male and female partners. Replicating prior work, preferences for most classically sex-differentiated traits (e.g., physical attractiveness, age) were found to be driven by one’s own sex. However, overall standards were found to be driven in large part by partner sex, with higher standards expressed for ideal male partners relative to ideal female partners. These results suggest that at least one aspect of mating psychology, mate preferences, is tied not only to one’s own sex but also to the sex of the romantic target being evaluated. More broadly, these results highlight the importance of more directly testing competing functional designs through which the same ultimate outcome may be proximately instantiated.

## Transparency

*Action Editor:* Carin Perilloux

*Editor:* Patricia J. Bauer

*Author Contributions*

**Ashley J. Coventry:** Conceptualization; Data curation; Formal analysis; Investigation; Methodology; Visualization; Writing – original draft; Writing – review & editing.

**Selina Mixner:** Conceptualization; Data curation; Methodology.

**Benjamin Gelbart:** Conceptualization; Data curation; Formal analysis; Investigation; Methodology; Writing – review & editing.

**Kathryn Walter:** Conceptualization; Data curation; Formal analysis; Investigation; Methodology; Writing – review & editing.

**Daniel Conroy-Beam:** Conceptualization; Data curation; Formal analysis; Funding acquisition; Investigation; Methodology; Project administration; Supervision; Visualization; Writing – review & editing.

**Tamsin German:** Conceptualization; Data curation; Funding acquisition; Investigation; Methodology; Project administration; Supervision; Writing – review & editing.

**Artificial Intelligence:** No AI-assisted technologies were used in this research or the creation of this article.

**Ethics:** This research received approval from the University of California, Santa Barbara Institutional Review Board.

**Open Practices:** Study 1. Preregistration: This study was not preregistered. Materials: The materials are publicly accessible at https://doi.org/10.17605/OSF.IO/AMX5V. Data: The data are publicly accessible at https://doi.org/10.17605/OSF.IO/AMX5V. Analysis scripts: The analysis scripts are publicly accessible at https://doi.org/10.17605/OSF.IO/AMX5V. Study 2. Preregistration: This study was not preregistered. Materials: The materials are publicly accessible at https://doi.org/10.17605/OSF.IO/AMX5V. Data: The data are publicly accessible at https://doi.org/10.17605/OSF.IO/AMX5V. Analysis scripts: The analysis scripts are publicly accessible at https://doi.org/10.17605/OSF.IO/AMX5V.
